# Reduced lifespan of erythrocytes in Dahl/Salt sensitive rats is the cause of the renal proximal tubule damage

**DOI:** 10.1038/s41598-020-79146-9

**Published:** 2020-12-16

**Authors:** Eri Manabe, Satoyasu Ito, Yoshiya Ohno, Toshiyuki Tanaka, Yoshiro Naito, Naoko Sasaki, Masanori Asakura, Tohru Masuyama, Masaharu Ishihara, Takeshi Tsujino

**Affiliations:** 1 Department of Cardiovascular and Renal Medicine, Hyogo College of Medicine, 1-1 Mukogawa-cho, Nishinomiya, Hyogo 663-8501 Japan; 2 Division of Pharmaceutical Therapeutics, Department of Pharmacy, School of Pharmacy, Hyogo University of Health Sciences, Kobe, Hyogo Japan; 3 Division of Immunobiology, Department of Pharmacy, School of Pharmacy, Hyogo University of Health Sciences, Kobe, Hyogo Japan; 4 Hoshigaoka Medical Center, Japan Community Health Care Organization, Hirakata, Osaka Japan

**Keywords:** Cardiovascular biology, Experimental models of disease

## Abstract

We studied the mechanisms of anemia and the influence of anemia on renal pathology in Dahl/Salt Sensitive (Dahl/SS) rat, a model of cardio-renal-anemia syndrome. Erythrocyte lifespan was shortened and associated with decreased hemoglobin level in the Dahl/SS rats given high-salt diet. Serum haptoglobin decreased, reticulocytes increased, and erythropoiesis in the bone marrow and extramedullary hematopoiesis in the spleen was markedly stimulated by increased serum erythropoietin in them. As a mechanism of hemolysis, we investigated the incidence of eryptosis, suicidal death of erythrocytes. Eryptosis was increased, and red blood cell-derived microparticles, small particle which are generated in hemolytic disease, were also increased in Dahl/SS rats fed with high-salt diet. Deposition of hemosiderin and mitochondrial morphologic abnormality, a sign of ferroptosis, in proximal renal tubules was associated with intravascular hemolysis. Treatment with deferasirox, an oral iron chelator, reduced the renal proximal tubular injury and the glomerular sclerosis in Dahl/SS rats fed with high-salt diet. In conclusion, reduced half-life of erythrocytes induced by hemolysis is the major cause of anemia in Dahl/SS rat. Iron accumulation induced by hemolysis causes renal proximal tubule injury and accelerates renal damage in this model.

## Introduction

With the aging of the population, the prevalence of cardiovascular disease is rising, and heart failure as the final stage adversely affects multiple organs, and results in a decrease in the quality of life. Patients with chronic heart failure (CHF) are complicated by anemia in about 30–60%^1–4^. The frequency of anemia increases, as heart failure becomes severe, and anemia is an independent poor prognostic factor of heart failure^5^. Since chronic kidney disease (CKD) was known to be associated with anemia and CHF, the concept of cardio-renal-anemia syndrome (CRAS) has been proposed^6,7^. Anemia can worsen not only cardiac function, but also renal function, and may cause more rapid progression of cardio-renal failure than in patients without anemia.

Cause of anemia in CRAS has been suggested to be multifactorial. It includes increased circulating plasma volume due to fluid retention^8^, decreased bone marrow hematopoietic response^9^, decreased erythropoietin secretion with CKD, iron absorption/use disorder^10,11^, nutrient deficiency due to decreased appetite, steroid metabolism abnormalities^12^, and effects of therapeutic drugs, etc. However, causes of anemia in CRAS are still largely unknown. It is important to elucidate the mechanism to establish the treatment of anemia in CRAS.

CHF is currently divided into 2 classes: i.e. heart failure with reduced ejection fraction (HFrEF) and heart failure with preserved ejection fraction (HFpEF)^13^. Anemia is more prevalent in patients with HFpEF than HFrEF^14^. Dahl/Salt Sensitive (Dahl/SS) rat is the most frequently used animal model of HFpEF^15^. We have previously reported that Dahl/SS rat is a model of CRAS^11,16–18^. We have also reported that abnormal iron metabolism in Dahl/SS rats was similar to that in patients with CHF^11^. Here we report the mechanisms of anemia in Dahl/SS rat. In the course of research, we found intravascular hemolysis was the major cause of anemia in Dahl/SS rats. We also report the role of hemosiderin accumulation in renal damage and effect of treatment with deferasirox (DFX), an oral iron chelator.

## Results

### Characteristics of Dahl/SS rats

**Figure 1:**
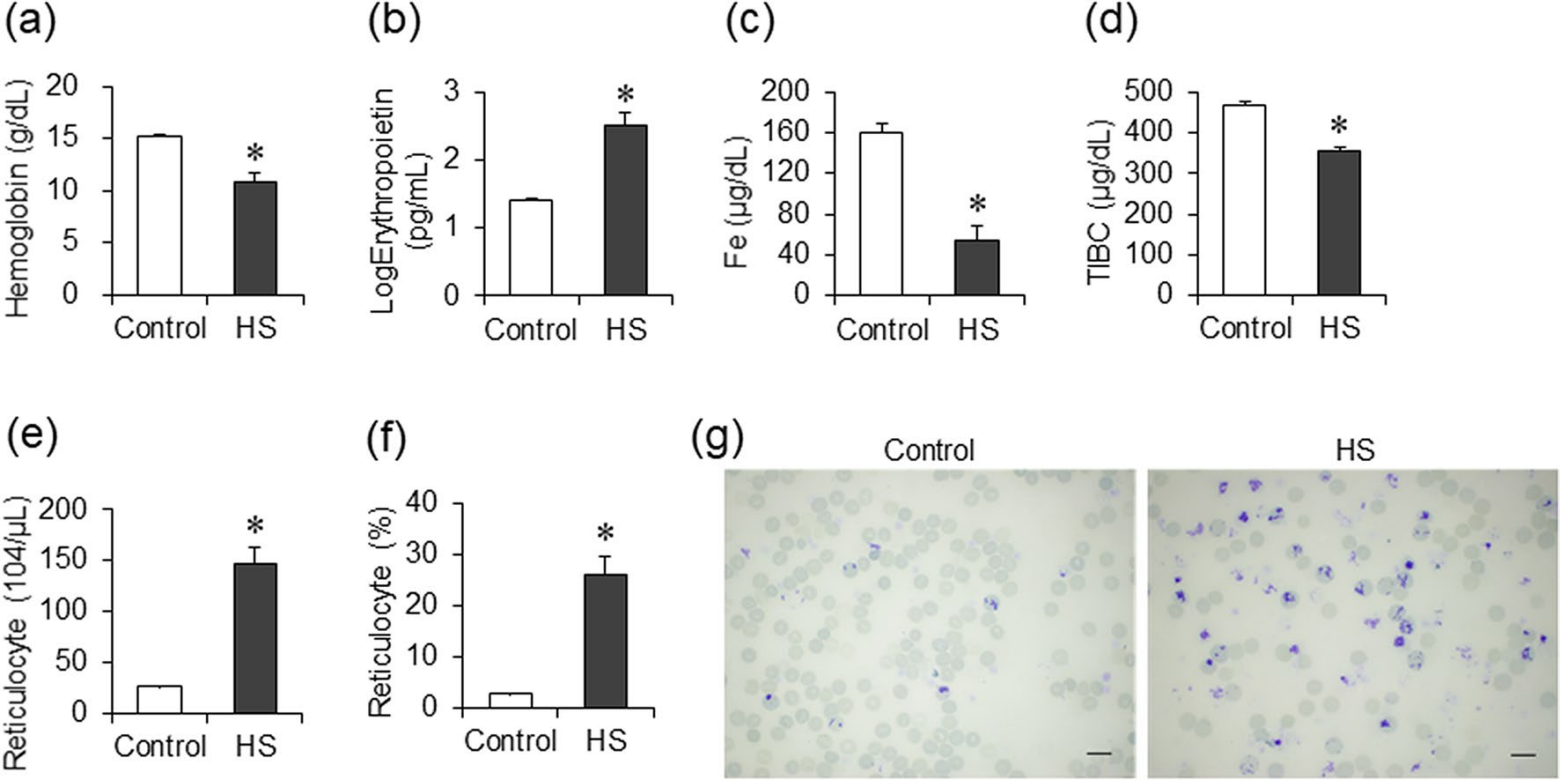
Characteristics of anemia in Dahl/SS rats. Comparison of hemoglobin (**a**), log-transformed serum erythropoietin (**b**), Fe (**c**), and total iron binding capacity (TIBC) (**d**) levels in the control (n = 7) and the HS (n = 8) groups at 15–17 weeks of age. The number (**e**) and ratio (**f**) of reticulocyte in the control (n = 7) and the HS (n = 9) groups. Supravital staining image of reticulocytes by new methylene blue (bars 10 μm) in the control and the HS groups (**g**). Statistical analysis was performed using unpaired Student’s t test by JMP Pro Version 14.2.0 software (SAS Institute Inc.). *p < 0.05 vs. the control group.

Characteristics of Dahl/SS rats in the control and the HS group are shown in Table 1. Compared to the control group, blood pressure and serum creatinine level increased, and the hemoglobin level decreased with increasing MCV (Table 1 and Fig. 1a) in the HS group. Decreased hemoglobin levels were accompanied by increased serum erythropoietin levels (Fig. 1b), decreased serum iron and total iron binding capacity (TIBC), and increased number of peripheral blood reticulocytes in the HS group (Fig. 1c–g). Increase in MCV and RDW-CV was suggested to reflect increased number of reticulocytes, which were larger than usual erythrocytes (Table 1).

**Table 1:** Physiologic and hematologic parameters at 15–17 weeks of age.

Parameter	Control	HS
Body weight (g)	419.4 ± 7.4	331.8 ± 11.3*
Systolic blood pressure (mmHg)	114.5 ± 5.5	196.5 ± 5.4*
Diastolic blood pressure (mmHg)	89.4 ± 5.5	162.1 ± 5.5*
Heart rate (bpm)	381.3 ± 11.9	489.6 ± 9.1*
Lung weight/tibia length (mg/mm)	38.5 ± 1.5	41.7 ± 1.5
LV weight/tibia length (mg/mm)	23.9 ± 0.6	34.4 ± 0.9*
Spleen weight/tibia length (mg/mm)	23.8 ± 0.4	55.0 ± 4.0*
Kidney weight/tibia length (mg/mm)	36.5 ± 1.6	48.4 ± 0.8*
Red blood cell (× 10^6^/μL)	9.00 ± 0.11	5.91 ± 0.45*
Hemoglobin (g/dL)	15.3 ± 0.2	10.9 ± 0.8*
Hematocrit (%)	45.4 ± 0.5	38.0 ± 2.0*
MCH (pg)	17.0 ± 0.1	18.4 ± 0.3*
MCV (fL)	50.5 ± 0.3	65.3 ± 2.2*
MCHC (g/dL)	33.6 ± 0.3	28.4 ± 0.7*
Reticulocyte (%)	2.9 ± 0.1	26.3 ± 3.4*
Reticulocyte (× 10^4^/μL)	26.1 ± 1.1	146.2 ± 17.3*
RDW-CV (%)	20.1 ± 0.3	23.4 ± 1.1*
Platelet (× 10^4^/μL)	77.0 ± 3.4	71.8 ± 4.3
Creatinine (mg/dL)	0.37 ± 0.01	0.66 ± 0.04*

*p < 0.05 versus the control group.

### Effects of serum erythropoietin levels on erythroid cells of the bone marrow and spleen

**Figure 2:**
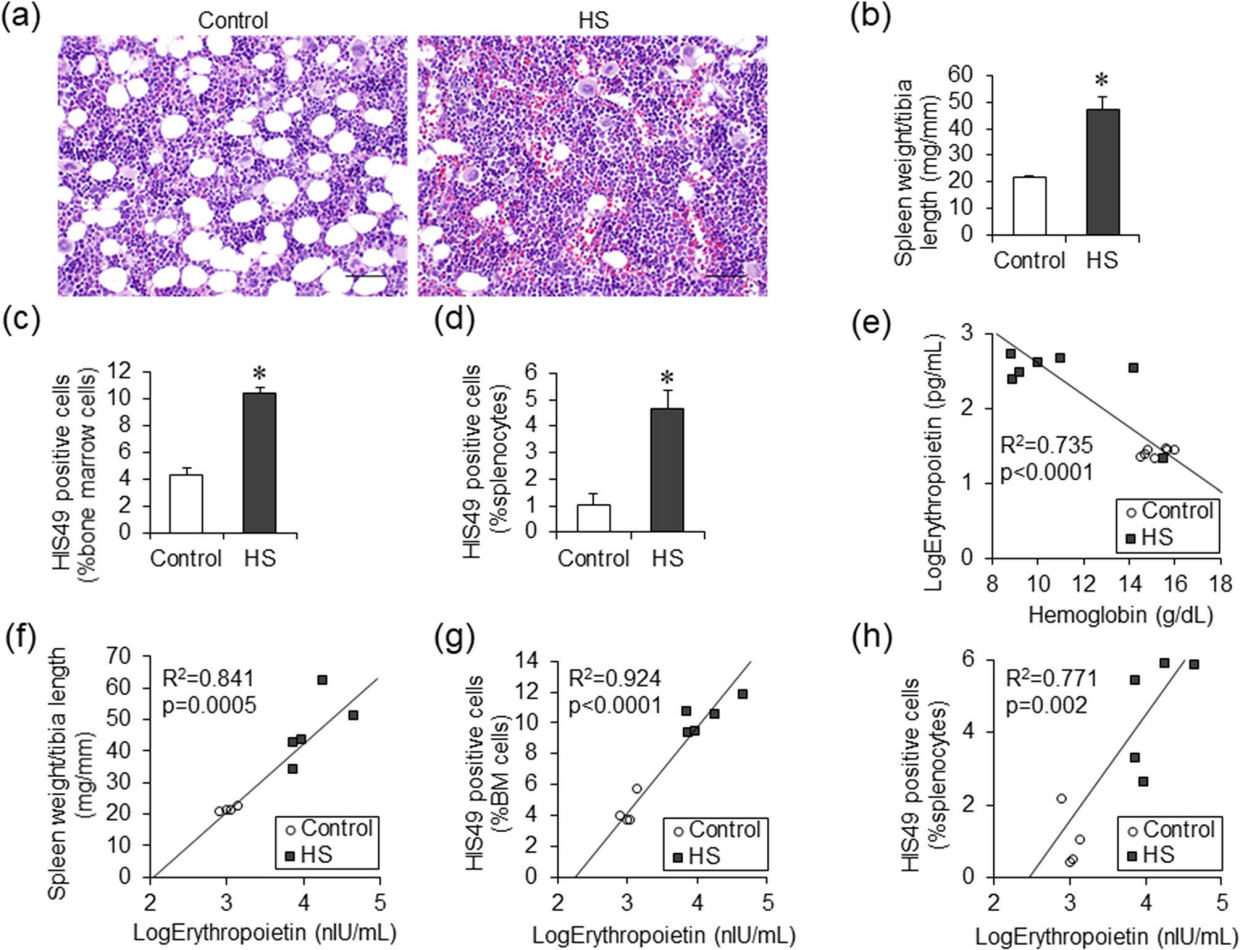
Bone marrow and splenic hematopoiesis in Dahl/SS rats. Hematoxylin and eosin staining of femur bone marrow in the control and the HS groups (**a**). (bars 50 μm) Spleen weight (**b**), the percentage of HIS49 positive cells in femur bone marrow cells (**c**) and splenocytes (**d**) in the control (n = 4) and the HS (n = 5) groups. The correlation of hemoglobin levels and serum erythropoietin levels in the control (n = 7) and the HS (n = 7) groups (**e**). The correlation of serum erythropoietin levels and spleen weight (**f**), the percentage of HIS49 positive cells in femur bone marrow cells (**g**), and those in splenocytes (**h**) in the control (n = 4) and the HS (n = 5) groups. Statistical analysis was performed using unpaired Student’s t test in (**b**), (**c**), (**d**). The associations between two parameters were analyzed using Pearson’s correlation coefficient in (**e**), (**f**), (**g**) and (**h**) by JMP Pro Version 14.2.0 software (SAS Institute Inc.). *p < 0.05 vs. the control group.

In hematoxylin and eosin staining of the femur bone marrow, lipid droplets were observed in the control groups, while nucleated cell density increased, and lipid droplets almost disappeared in the HS groups (Fig. 2a). In addition, spleen weight was increased in the HS groups as compared with the control groups (Fig. 2b). To determine the hematopoietic potential in bone marrow or spleen in the HS groups, flow cytometry assays were performed using HIS49, a surface marker of erythroid cells in rats. Compared to the control groups, the proportion of HIS49-positive cells in both bone marrow and spleen nucleated cells increased in the HS groups (Fig. 2c,d). Erythropoietin is known to act on erythroid progenitor cells and erythroblasts to increase the number of erythrocytes. Serum erythropoietin levels increased in the HS group (Fig. 1b) and correlated negatively with hemoglobin levels, which suggested that erythropoietin secretion was enhanced by anemia for compensation (Fig. 2e). Positive correlation between spleen weight and serum erythropoietin concentration suggested enhanced extramedullary hematopoiesis in the spleen (Fig. 2f). Positive correlation between serum erythropoietin levels and percentage of bone marrow and splenic HIS49-positive cells confirmed that erythropoiesis in the bone marrow and extramedullary hematopoiesis in the spleen was markedly stimulated in response to elevated serum erythropoietin in the HS group. (Fig. 2g,h).

### Half-life of erythrocytes reduced under high-salt diet

**Figure 3:**
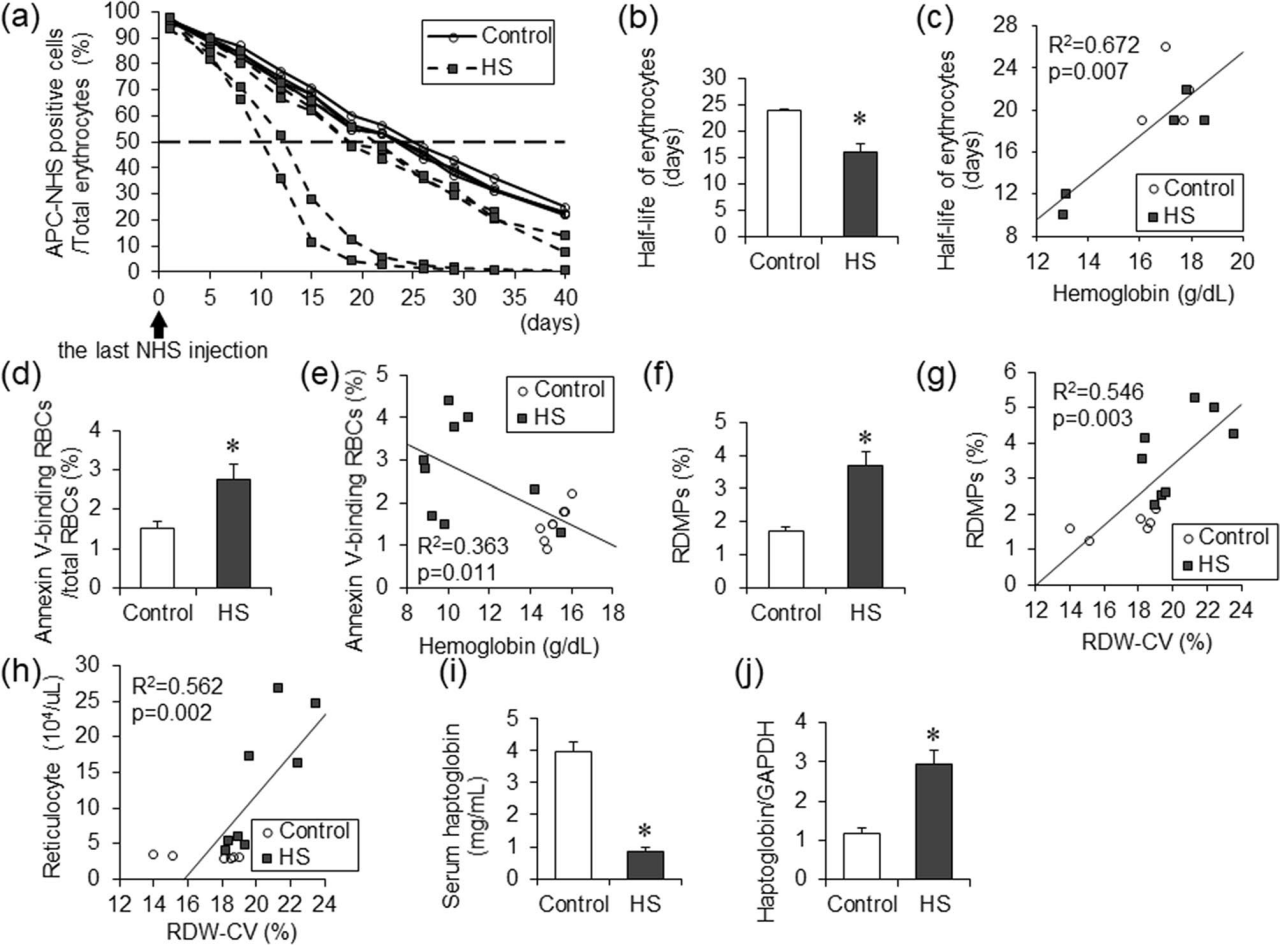
Half-life of erythrocytes in Dahl/SS rats. The decay curves of erythrocytes were expressed as the percentage of APC-NHS positive cells in total erythrocytes after the last Sulfo-NHS-Biotin injection in the control (n = 4) and the HS (n = 5) groups (**a**). The half-life of erythrocytes in the control (n = 4) and the HS (n = 5) groups (**b**). The correlation between the hemoglobin level and the half-life of erythrocytes in the control (n = 4) and the HS (n = 5) groups (**c**). The percentage of annexin V-binding erythrocytes in total erythrocytes in the control (n = 8) and the HS (n = 9) groups (**d**). The correlation between the hemoglobin level and the percentage of annexin V-binding erythrocytes in total erythrocytes in the control (n = 8) and the HS (n = 9) groups (**e**). The percentage of RDMPs in total erythrocytes in the control (n = 6) and the HS (n = 8) groups (**f**). The correlation of RDW-CV and the proportion of RDMPs in total erythrocytes in the control (n = 6) and the HS (n = 8) groups (**g**). The correlation between RDW-CV and the number of reticulocytes in the control (n = 6) and the HS (n = 8) groups (**h**). The serum haptoglobin level in the control (n = 8) and the HS (n = 9) groups (**i**). The haptoglobin/GAPDH mRNA ratio in the liver in the control (n = 8) and the HS (n = 5) groups (**j**). Statistical analysis was performed using unpaired Student’s t test in (**b**), (**d**), (**f**), (**i**), and (**j**). The associations between two parameters were analyzed using Pearson’s correlation coefficient in (**c**), (**e**), (**g**) and (**h**) by JMP Pro Version 14.2.0 software (SAS Institute Inc.). *p < 0.05 vs. the control group.

In the HS group, anemia was accompanied by increased hematopoiesis in bone marrow and spleen, suggesting a shortening of the erythrocyte lifespan. To investigate the half-life of erythrocytes in the HS group, flow cytometry assays were performed. Twelve-week-old Dahl/SS rats in the control and HS groups were injected with Sulfo-NHS-Biotin to label erythrocytes with biotin in peripheral blood. Blood were drawn into tubes containing the day after the injection (day 1) and then twice a week to measure the lifespan of erythrocytes. Compared to the control group, the half-life of erythrocytes was shortened in the HS group (Fig. 3a,b). In addition, there was a significant correlation between hemoglobin levels and half-life of erythrocytes (Fig. 3c). These findings suggested that hemolysis was a cause of anemia.

As a mechanism of hemolysis, we investigated the incidence of eryptosis, suicidal death of erythrocytes (Fig. 3d,e). The ratio of Annexin-V positive erythrocytes to total erythrocytes was larger in the HS group than the control group, and negatively associated with hemoglobin level.

Red blood cell-derived microparticles (RDMPs) are small particle which are generated in hemolytic disease. RDMPs contain hemoglobin and induce vascular damage by scavenging nitric oxide. The ratio of RDMPs to total erythrocytes was larger in the HS group than the control group (Fig. 3f). Positive correlation between the ratio of RDMPs with RDV-CV suggested the presence of fragmented red blood cell contribute to the increased RDW-CV in the HS group (Fig. 3g)^19^. Increase RDW-CV was also correlated to increased number of reticulocytes, which were larger than usual erythrocytes (Fig. 3h).

Compared to the control group, serum concentration of haptoglobin decreased in the HS group although the mRNA level of haptoglobin in the liver was increased, suggesting that increased consumption of haptoglobin induced by hemolysis was the cause of decreased serum haptoglobin concentration (Fig. 3i,j).

### Intravascular hemolysis causes shortening of erythrocyte half-life

**Figure 4:**
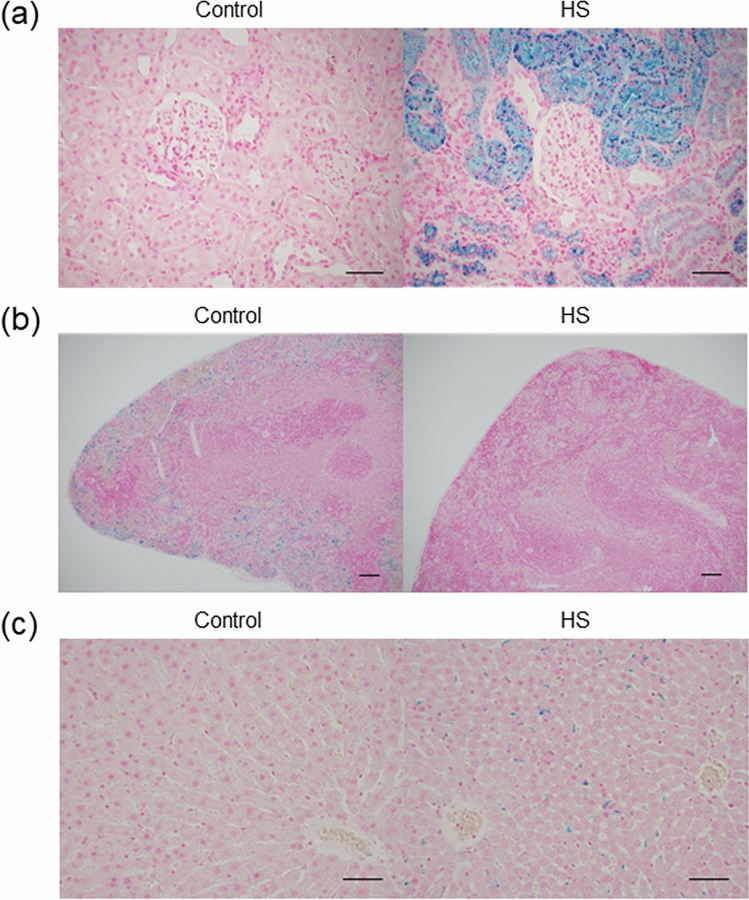
Tissue distribution of hemosiderin in Dahl/SS rats. Histological analysis of kidney (**a**), spleen (**b**) and liver (**c**) with Berlin Blue staining in the control and the HS groups. (**a**, **c**; bars 50 μm, **b**; bars 100 μm).

Depending on the location of erythrocyte destruction, hemolysis is divided into extravascular hemolysis and intravascular hemolysis. In extravascular hemolysis, erythrocytes are trapped and phagocytosed by macrophages, and hemoglobin is metabolized. In intravascular hemolysis, most of the hemoglobin released into the blood vessel forms immediately haptoglobin-hemoglobin complexes and is metabolized after being transported to the liver, while the remaining hemoglobin is filtered at the glomerulus of the kidney, reabsorbed by the renal tubular epithelium and metabolized to hemosiderin. To investigate the tissue distribution of hemosiderin, which is the ferric ion, Berlin Blue staining was performed. In comparison with the control group, the deposition of hemosiderin was increased in the proximal tubule of kidney and the liver (Fig. 4a,c) but decreased in the spleen (Fig. 4b) in the HS group. These findings suggested that intravascular hemolysis occurred in the HS group.

### Effects of iron chelation on renal pathophysiology

**Figure 5:**
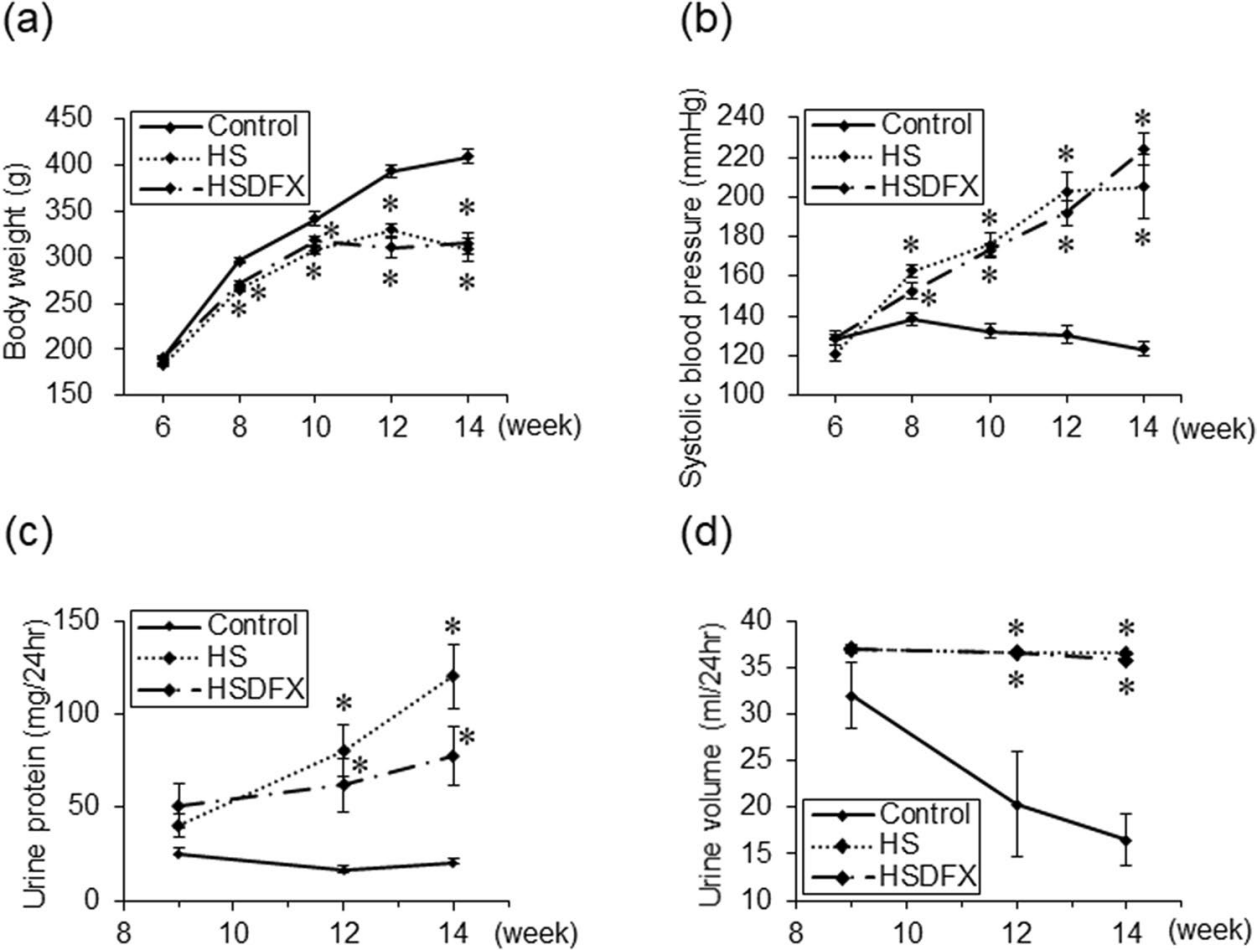
Changes in physiologic parameters and urine-protein by administration of deferasirox. Body weight (**a**), Systolic blood pressure (**b**), Urine volume for 24 h (**c**), and Urine-protein for 24 h (**d**) in the control (n = 6), the HS (n = 6) and the HSDFX (n = 4) groups. Statistical analysis for each time-point was performed using one-way analysis of variance followed by Tukey–Kramer test in (**a**), (**b**), (**c**), and (**d**) by JMP Pro Version 14.2.0 software (SAS Institute Inc.). *p < 0.05 vs. the control group.

We hypothesized that accumulation of hemosiderin in the tubules might accelerate the renal damage in the HS group. To ameliorate the deposition of hemosiderin into the renal proximal tubular epithelial cells caused by intravascular hemolysis, iron chelator DFX was administered by gavage to the Dahl/SS rats fed with high-salt diet. Blood pressure, hemoglobin level, serum iron and creatinine, and urine protein in the HSDFX Group were similar to those in the HS Group. (Table 2 and Fig. 5a–d).

**Table 2:** Physiologic and hematologic parameters at 14–15 weeks of age.

Parameter	Control	HS	HSDEF
Body weight (g)	417.5 ± 8.1	310.2 ± 16.8*	319.7 ± 19.4*
Systolic blood pressure (mmHg)	123.3 ± 3.2	205.1 ± 16.2*	224.0 ± 8.1*
Diastolic blood pressure (mmHg)	100.8 ± 2.8	170.7 ± 15.5*	183.0 ± 9.4*
Heart rate (bpm)	414.4 ± 13.3	446.2 ± 32.5	451.5 ± 19.6
Lung weight/tibia length (mg/mm)	36.5 ± 0.8	36.2 ± 1.0	36.7 ± 1.5
LV weight/tibia length (mg/mm)	22.0 ± 0.3	30.1 ± 0.6*	31.2 ± 1.2*
Spleen weight/tibia length (mg/mm)	24.1 ± 1.1	45.6 ± 3.5*	43.4 ± 8.1^†^
Kidney weight/tibia length (mg/mm)	36.2 ± 0.9	42.9 ± 1.9*	39.9 ± 1.9
Red blood cell (× 10^6^/μL)	9.07 ± 0.65	6.34 ± 0.51*	5.79 ± 1.04*
Hemoglobin (g/dL)	15.7 ± 1.3	11.5 ± 0.8*	10.2 ± 1.9*
Hematocrit (%)	45.8 ± 2.3	39.8 ± 1.8	34.7 ± 4.2*
MCH (pg)	17.3 ± 0.2	18.3 ± 0.3	17.5 ± 0.6
MCV (fL)	50.9 ± 1.0	63.7 ± 2.3*	61.8 ± 3.7*
MCHC (g/dL)	34.1 ± 0.9	28.8 ± 0.7*	28.7 ± 1.9*
Reticulocyte (%)	3.0 ± 0.1	24.3 ± 4.1*	25.0 ± 8.5*
Reticulocyte (× 10^4^/μL)	27.2 ± 2.6	145.0 ± 21.7*	120.5 ± 31.4*
RDW-CV (%)	20.5 ± 0.8	25.1 ± 1.6*	24.3 ± 0.9
Platelet (× 10^4^/μL)	102.3 ± 6.7	96.6 ± 11.2	97.9 ± 22.8
Creatinine (mg/dL)	0.35 ± 0.02	0.68 ± 0.08*	0.61 ± 0.07*
Fe (μg/dL)	170.0 ± 12.5	77.0 ± 18.6*	73.5 ± 22.6*
TIBC (μg/dL)	446.2 ± 9.8	357.7 ± 13.9*	365.0 ± 16.9*
UIBC (μg/dL)	276.2 ± 19.9	280.7 ± 17.0	291.5 ± 34.0
Total-billirubin (mg/dL)	0.07 ± 0.01	0.08 ± 0.03	0.11 ± 0.01*
Indirect-billirubin (mg/dL)	0.05 ± 0.01	0.06 ± 0.02	0.09 ± 0.01*
Urine volume (ml/24 h)	16.5 ± 2.8	36.6 ± 0.2*	35.8 ± 0.2*
Urine protein (mg/24 h)	20.5 ± 2.4	120.1 ± 17.3*	77.6 ± 15.8*
Urine protein/creatinine	1.4 ± 0.2	23.6 ± 3.7*	17.6 ± 4.1*

*p < 0.05; vs. the control group, ^†^*p* < 0.05; the HSDFX versus the control group.

**Figure 6:**
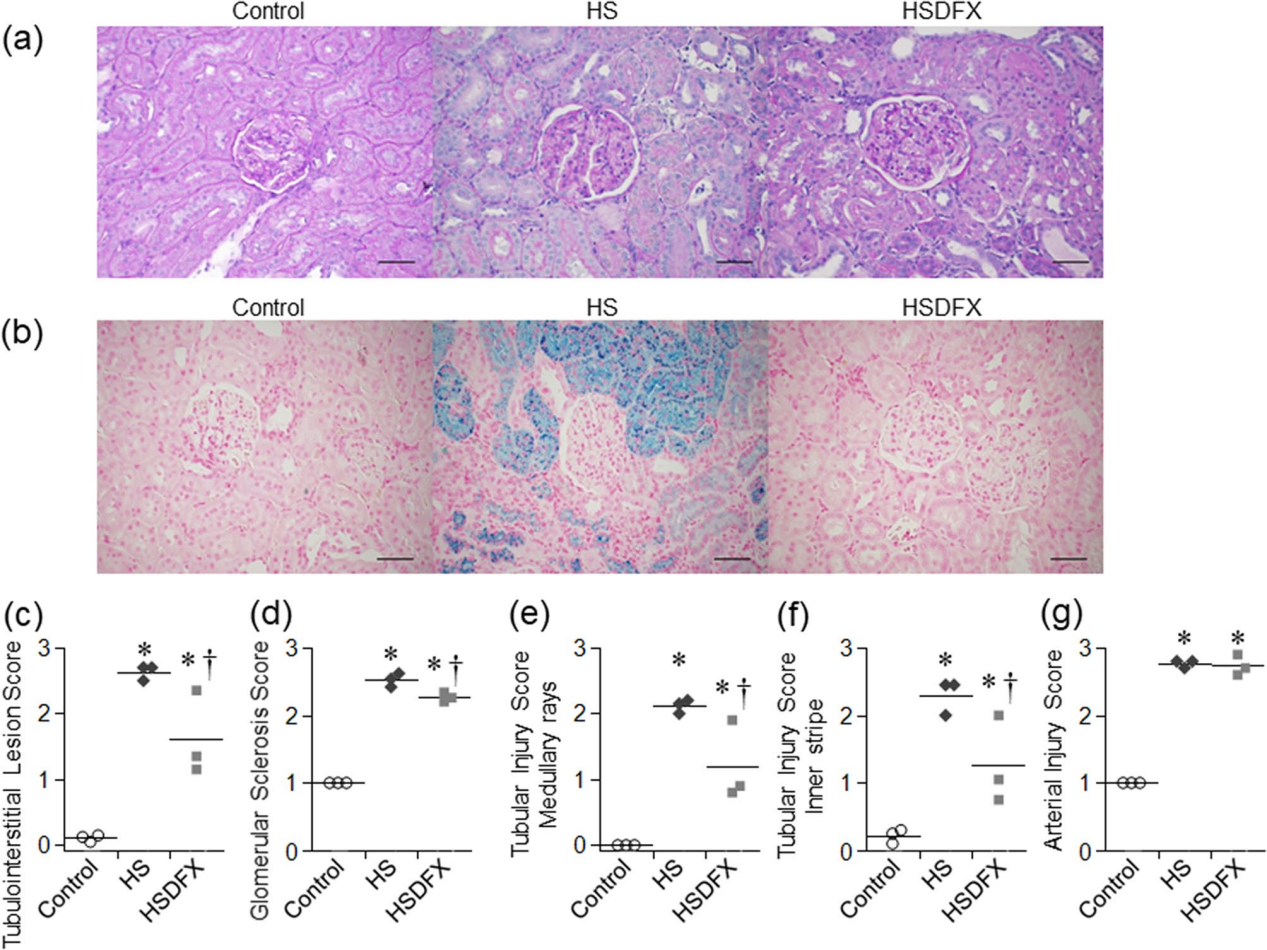
Histological analysis of kidney in Dahl/SS rats. (**a**) Representative PAS staining of the renal cortex in the control, the HS and the HSDFX groups (bars 100 μm). (**b**) Representative Berlin blue staining of the renal cortex in the control, the HS and the HSDFX groups (bars 100 μm). As histological analysis of kidney, tubulointerstitial lesion score (**c**), glomerular sclerosis score (**d**), tubular injury score in the medullary rays (**e**) and the inner stripe (**f**), and arterial injury score (**g**) are shown. Statistical analysis was performed using one-way analysis of variance followed by Tukey–Kramer test in (**c**), (**d**), (**e**), (**f**), and (**g**) by JMP Pro Version 14.2.0 software (SAS Institute Inc.). *p < 0.05 vs. the control group, ^†^*p* < 0.05 vs. the HS group.

We investigated whether DFX improved renal histologic findings. Berlin Blue staining revealed that deposition of hemosiderin in renal proximal tubular epithelial cells in the HS group was decreased by DFX treatment (Fig. 6a,b). Compared to the HS Group, the Tubular Injury Score and the Glomerular Sclerosis Score were reduced in the HSDFX Group (Fig. 6c–f), but the Arterial Injury Score did not improve (Fig. 6g).

**Figure 7:**
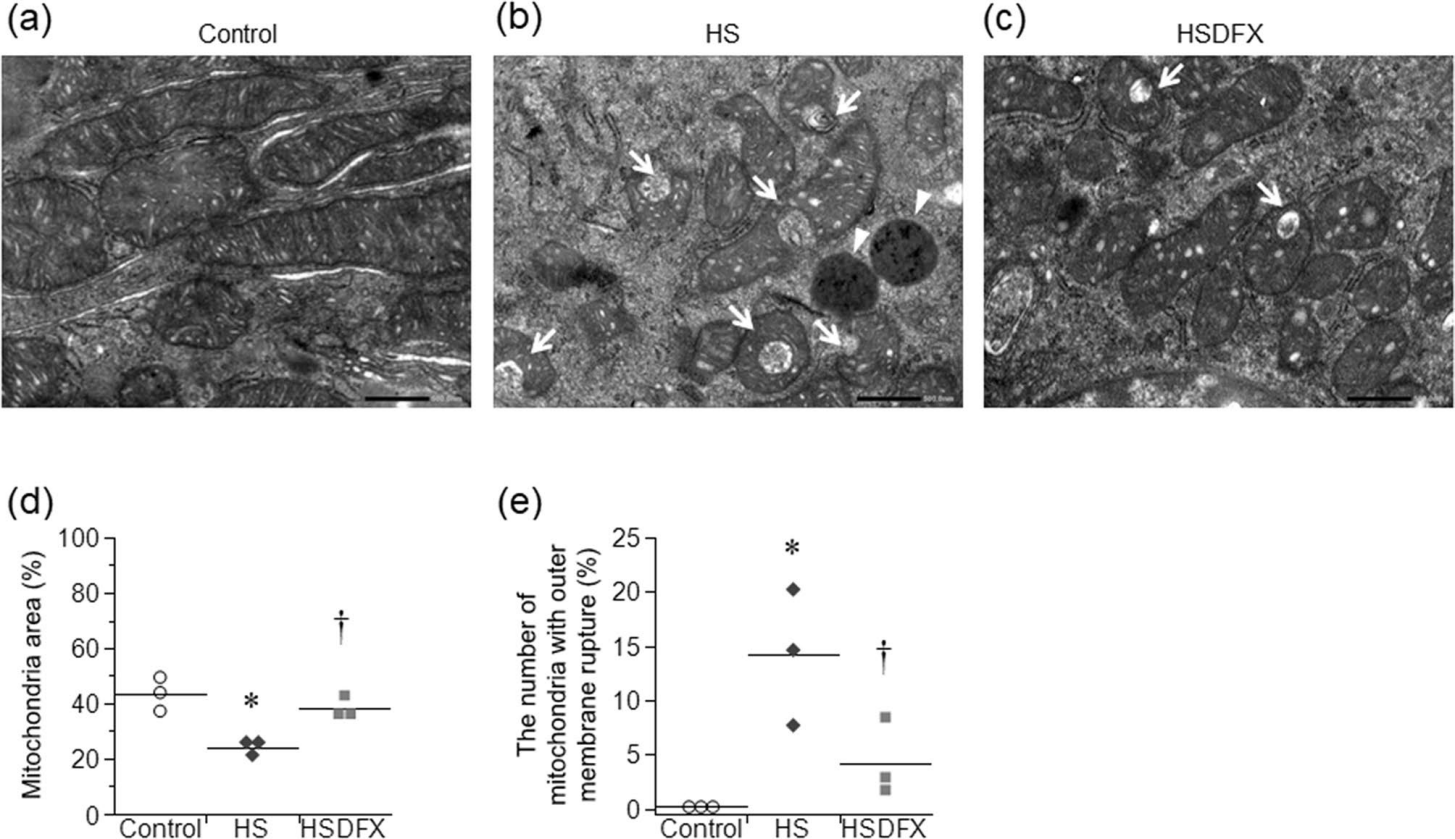
Mitochondrial damage in the renal proximal tubular epithelial cells. The electron micrographs show the mitochondria in the renal proximal tubular epithelial cells in the control group (**a**), the HS group (**b**), and the HSDFX group (**c**) (bars 500 nm). Panel (**d**) shows the percentage of mitochondria area in cytoplasm. Panel (**e**) shows the percentage of mitochondria with outer membrane rupture in the total number of mitochondria. (n = 3 for each group, Arrow: outer mitochondrial membrane rupture, Arrowhead: lysosome). Statistical analysis was performed using one-way analysis of variance followed by Tukey–Kramer test in (**d**) and (**e**) by JMP Pro Version 14.2.0 software (SAS Institute Inc.). *p < 0.05 vs. the control group, ^†^*p* < 0.05 vs. the HS group.

For further investigation whether hemosiderin deposition causes renal proximal tubular epithelial cells damage, the electron microscopy was performed. In the HS group, lysosomes containing dense bodies suggesting iron granules were found in the renal tubular cytoplasm, but almost disappeared in the HSDFX group (Fig. 7a–c). Compared to the control group, mitochondrial size was small, and mitochondria with outer membrane rupture, a sign of ferroptosis, was found in the HS Group. In the HSDFX group, both were reduced compared to the HS group (Fig. 7a–e).

## Discussion

This study showed that anemia in Dahl/SS rats fed with high-salt diet was caused by reduced half-life of erythrocytes in peripheral blood. First, erythropoiesis of Dahl/SS rats was not impaired. The number of reticulocytes, a marker of hematopoietic ability, and erythroid cells in bone marrow and spleen increased. In this model, EPO secretion failure was not observed though the serum creatinine concentration slightly increased, and the increase in the serum EPO concentration was related to the decrease in hemoglobin level and the increase in erythroid cells. These findings suggested that hematopoiesis of erythroid cells in bone marrow and spleen of Dahl/SS rats is enhanced by increased serum EPO level. Serum EPO concentration in patients with heart failure reflects hypoxia in the kidneys and enhancement of the RA system, so it is one of the poor prognostic factors^20^. Serum EPO level correlates reticulocyte count in renal dialysis patients, and the increase in reticulocyte count is thought to be a compensatory change by EPO. If similar reaction also occurs in patients with heart failure with anemia, reticulocyte count could be a marker of poor prognosis^21^. Second, in Dahl/SS rats, directly measured erythrocyte half-life was shortened, which correlated with the hemoglobin level. Young reticulocyte membranes produced under stress hematopoiesis such as EPO increase are less deformable, unstable to mechanical stimuli and prone to hemolysis. Therefore, not all erythrocytes produced by stress hematopoiesis become mature erythrocytes^22–24^. It has also been reported that erythrocyte clearance from peripheral blood is accelerated in pathological conditions in which oxidative stress is elevated, such as chronic inflammation models and chronic renal failure/hemodialysis patients^25^. Thus, reduced half-life of erythrocytes is the major cause of anemia in this model.

Next, we examined the cause of reduced half-life of erythrocytes in Dahl/SS rats. Reduced serum haptoglobin level and increased accumulation of hemosiderin in the kidney suggested that intravascular hemolysis causes reduced half-life of erythrocytes. The presence of juvenile reticulocytes, produced under stress hematopoiesis, also induced intravascular hemolysis. On the other hand, increased eryptosis, suicidal death of erythrocytes characterized by cell contraction and exposure of phosphatidylserine (PS) on the cell surface, also observed in this model^26^. Eryptotic erythrocytes, as well as apoptotic cells, bind to the PS receptor of macrophages, rapidly be removed, stay in the spleen for a long time, and cause splenomegaly^27^. In the past reports, splenomegaly was observed during activation of eryptosis in mice, and contributes to the improvement of erythrocyte clearance. Splenomegaly was also observed in this model and patients with CHF. Thus, extravascular hemolysis accelerated by eryptosis should also be considered as a cause of shortening of erythrocyte lifespan in this model^21^. One might think increase in eryptosis is too modest to induce anemia. Annexin-V labeled erythrocytes are eliminated quickly from the circulation. Therefore, it is considered that only a slight increase in circulating PS-exposed erythrocytes is observed. The mechanisms how salt loading induces eryptosis in Dahl/SS rats are currently unknown. Many pathways such as oxidative stress, energy deficiency/dysregulation, and osmotic balance are known to trigger eryptosis, while nitric oxide (NO) has been shown to inhibit it^26^. Changes in these factors can occur in this model. Especially, enhanced reactive oxygen species (ROS) production in renal outer medulla was reported to reduce NO tubular-vascular cross-talk and induce salt-sensitive hypertension in Dahl salt-sensitive rats^28^. Such an imbalance between ROS and NO might occur in red blood cell and trigger eryptosis. Further studies are necessary. In addition, eryptosis can form a vicious cycle of intravascular and extravascular hemolysis. Even when slight hemolysis occurs, free hemoglobin scavenges nitric oxide, induces vascular endothelial dysfunction and vasomotor disorder, activates the vascular endothelial adhesion factor, and finally cause inflammation and enhanced coagulation^29–36^. Moreover, endothelial cells to which eryptotic erythrocytes adhere are affected to induce microcirculation trouble and organ damage.

Furthermore, RDMPs increased in the peripheral blood in Dahl/SS rats fed with high-salt diet. RDMPs are formed by conditions of preserved erythrocytes, or diseases such as hemolytic anemia and sickle cell disease, in which intravascular hemolysis occur. RDMPs are supposed to be formed as a result of intravascular hemolysis in this model. RDMPs are microparticles that contain hemoglobin and scavenge nitric oxide and impair endothelial-dependent vasodilation because these particles are much smaller than erythrocytes and enter the cell-free zone of blood vessels^37,38^. In addition, free Hb has been shown to induce hypertension, which was prevented by haptoglobin, which trap and remove hemoglobin from circulation^39,40^. RDMPs may contribute to hypertension and organ damage in Dahl/SS rats fed with high-salt diet.

In intravascular hemolysis, most of the hemoglobin released into the blood vessel forms immediately haptoglobin-hemoglobin complexes and is metabolized after being transported to the liver, while the remaining hemoglobin is filtered at the glomerulus of the kidney, reabsorbed by the renal tubular epithelium and metabolized to hemosiderin. We examined whether hemosiderin accumulation accelerate renal damage in this model. Deferasirox (DFX) is an oral iron chelator and have a selective and high affinity for Fe^3+^. DFX-Fe complexes combined in a dose-dependent manner are excreted via the bile from the body. Treatment with DFX effectively prevented accumulation of hemosiderin and ameliorated renal damage in this model. Thus, iron overload to renal proximal tubular epithelial cell was thought to induce tubular injury. As a mechanism of tubular injury, we examined whether ferroptosis occurred in proximal tubules. Ferroptosis is a recently recognized form of cell death and has features different from apoptosis, necrosis and autophagy, three classical forms of cell deaths^35,36,38–47^. Ferroptosis is induced by iron-dependent generation of reactive oxygen species and accumulation of peroxidized lipids. Because a lack of convenient marker of ferroptosis, it is difficult to judge the incidence of ferroptosis in vivo. However, decreased mitochondrial size and outer mitochondrial membrane rupture, that are typical findings of ferroptosis, were ameliorated with decreased hemosiderin accumulation by iron chelator DFX. These findings suggested that ferroptosis occurred in renal proximal epithelial cells in this model. However, the effect of DFX was limited in Dahl/SS rats fed with high-salt diet. Though tubular injury was ameliorated by the treatment with DFX, improvement in glomerulosclerosis was marginal and decrease in urine protein and serum creatinine did not reach statistical significance. Because DFX treatment did not lower blood pressure, deleterious effect of hypertension on renal pathology was not fully prevented. Clinical utility of iron chelators for CRAS remained to be elucidated.

We found that hemolysis is the major cause of anemia and iron accumulation induced by hemolysis accelerates renal damage in Dahl/Salt sensitive rats. These findings suggest that prevention of hemolysis could be a new strategy of treating anemia in CRAS. Our findings suggested that eryptosis is one of the mechanisms of hemolysis in this model. Many triggers of eryptosis have been reported^26^. However, it is unknown how to inhibit eryptosis except for avoiding triggers. Development of new strategy for suppressing eryptosis may be useful for the treatment of anemia in CRAS.

There are several limitations in this study. First, contribution of ferroptosis to the renal damage was not completely proved. Because a lack of convenient marker, it is difficult to prove ferroptosis in vivo, especially in rats. Further studies are necessary. Second, intravascular hemolysis may not be the only source of iron accumulation in renal proximal tubule. Iron could be uptaken through transferrin receptor into cells^48^. Other mechanisms of iron accumulation to proximal tubule should be examined. Third, it is unknown how this model represents patients with CRAS. It is difficult to measure half-life of erythrocytes in patients with CHF. However, RDW-CV, which could reflect increased number of reticulocytes, has been shown to correlate positively with serum EPO levels and established as a poor prognostic marker of CHF^49^. Because etiology of anemia in CRAS is multifactorial, this model may represent CHF patients who have higher EPO levels than expected from hemoglobin levels, who are known to have poorer prognosis than CHF patients who have lower EPO levels than expected from hemoglobin levels^20^. Fourth, it was difficult to confirm that anemia in this model was developed due to heart failure because Dahl/SS rats are complicated with severe hypertension. It is known that malignant hypertension induces thrombotic microangiopathy (TMA)^50^. The clinical features of TMA include microangiopathic hemolytic anemia, thrombocytopenia, and organ injury. We did not think anemia of Dahl/SS rats was induced by TMA because we could not find thrombocytopenia in DS rats (Table 1). However, anemia of Dahl/SS rats may be an atypical variation of TMA. We should confirm our data in a HFpEF model without hypertension. However, such a model is not currently available as far as we know. Further studies are needed. Fifth, we have not performed precise functional validation of cardiac function such as LV pressure–volume loops in this study. Thus, we could not definitely conclude that Dahl/SS rats at 15 weeks of age were complicated with heart failure. We have reported that hemoglobin was significantly lower in the HS group than the control group at 14 weeks of age when high-salt diet was started at 6 weeks of age in Dahl/Salt sensitive rats^16^. We could not find increased lung weight in the HS group at 15–17 weeks of age in this study. However, normal lung weight does not necessarily mean the normal cardiac function. It is reasonable to assume that there exists mild diastolic heart failure at 15–17 weeks of age because we confirmed increased lung weight and echocardiographic parameters consistent with HFpEF at 18 weeks of age^16^.

In conclusion, reduced half-life of erythrocytes induced by intravascular and extravascular hemolysis is the major cause of anemia in Dahl/Salt sensitive rats. Iron accumulation induced by hemolysis causes renal proximal tubule injury and accelerates renal damage in this model.

## Methods

### Animal experiments

For all experiments, male Dahl/Salt Sensitive (Dahl/SS) rats (DIS/EisSlc, formerly named Dahl-Iwai S) were purchased from Japan SLC, Inc. (Shizuoka, Japan). Five-week-old rats fed a normal salt diet (0.3% NaCl; Oriental Yeast Co., ltd., Tokyo, Japan) and were allowed to acclimatize for a week. Rats were housed in a light-controlled room under a 12 h light–dark cycle and were allowed access to food and water ad libitum. At 6 weeks of age, they were randomly divided into 3 groups: the control group, the high-salt (HS) group, and the high-salt + deferasirox (HSDFX) group. The HS and HSDFX groups were given a high-salt diet (8%NaCl) from 6 weeks of age. The HSDFX group was orally administered DFX (200 mg/kg/day) for five days a week from 10 to 15 weeks of age. The systolic blood pressure (SBP) was measured using a noninvasive tail-cuff method every 2 weeks. All our experimental procedures received approval from the Animal Research Committee of Hyogo University of Health Sciences. This study was performed in accordance with Guidelines for proper conduct of animal experiments by the Science Council of Japan^51^.

### Hematologic and biochemical parameters

Whole blood was analyzed within 24 h from sampling, and serum and urine were stored at − 80 °C. Blood cell analysis, including reticulocyte count analysis, was performed with a blood cell analyzer XT-2000iV (Sysmex, Kobe, Japan) optimized for rats. A smear was prepared for a part of the whole blood and ultravital staining was performed. Serum levels of creatinine, total-bilirubin, indirect-bilirubin, iron, total iron binding capacity, erythropoietin and haptoglobin were analyzed. Erythropoietin and haptoglobin were measured by ELISA using Rat EPO ELISA Kit (BioLegend, San Diego, CA, USA) and Haptoglobin Rat ELISA kit (Abcam, Cambridge, UK). Urine sample was collected for 24 h, and protein and creatinine were measured.

### Half-life of erythrocytes

At 12 weeks of age, Dahl/SS rats in the control and the HS groups were injected with N-Hydroxysulfosuccinimido-biotin (Sulfo-NHS-Biotin; ApexBio, Houston, TX, USA), which was water-soluble biotinylation reagent to attach biotin to primary amine, into the tail vein on three consecutive days (40 μg/g body weight/3 days). Blood were drawn into tubes containing ethylenediaminetetraacetic acid dipotassium salt (EDTA-2K) the day after the injection (day 1) and then twice a week. To detect biotinylated erythrocytes, washed one million of erythrocytes were incubated with allophycocyanin (APC)-conjugated streptavidin (BD Biosciences, Tokyo, JAPAN) at 4 °C for 30 min in the dark, and washed twice in 0.2% BSA/PBS. The proportion of biotinylated erythrocytes in total erythrocytes was measured by flow cytometry (FACS Aria II, BD Biosciences, Tokyo, JAPAN).

### The proportion of erythroid cells

The proportion of erythroid cells in bone marrow cells and splenocytes was examined by flow cytometry analysis. Bone marrow cells were flushed out of femurs, and splenocytes were mechanically isolated from spleen. These cells were suspended in PBS/EDTA-2K. After hemolysis with tris-buffered ammonium chloride, nucleated cells were incubated with biotin-conjugated anti-rat HIS49 antibody (BD Biosciences, Tokyo, JAPAN) at 4 °C for 60 min. The cells were washed twice in 0.2% BSA/PBS and incubated with APC-conjugated streptavidin (BD Biosciences, Tokyo, JAPAN) at room temperature for 30 min in the dark. Dead cells were excluded with using 4′,6-diamidino-2-phenylindole. The proportion of erythroid cells in nucleated cells was measured by flow cytometry.

### The proportion of eryptotic erythrocytes

Blood were drawn from abdominal aorta into tubes containing EDTA-2K. To detect erythrocyte phosphatidylserine exposure, washed one million of erythrocytes were incubated with fluorescein isothiocyanate (FITC)-conjugated annexin V (BioLegend, San Diego, CA, USA) for 15 min at 4 °C in the dark. The proportion of annexin V positive erythrocytes in total erythrocytes was measured by flow cytometry.

### The proportion of red blood cell-derived microparticles

The proportion of red blood cell-derived microparticles (RDMPs) in peripheral blood was examined by flow cytometry analysis^37,52^. Blood were drawn into heparin tubes. Three million of erythrocytes were incubated with phycoerythrin (PE)-glycophorin A antibody (Abcam, Cambridge, UK) and APC-AnnexinV (BioLegend, San Diego, CA, USA) for 30 min at 4 °C in the dark. The proportion of RDMPs in glycophorin A-positive cells was measured using histograms of forward scatter and fluorescence intensity.

### Quantification of mRNA

Total RNA was extracted from liver using TRIzol reagent (Thermo Fisher Scientific, Tokyo, Japan) according to the manufacturer’s manual. Total RNA was reverse transcribed into cDNA using High Capacity cDNA reverse Transcription kit (Thermo Fisher Scientific, Tokyo, Japan) on GeneAmp PCR System 9700 (Thermo Fisher Scientific, Tokyo, Japan). Real-time polymerase chain reaction quantification of transcripts was performed with TaqMan Gene Expression Master Mix and TaqMan Gene Expression Assays (Applied Biosystems) on StepOnePlus Real-Time PCR Systems (Thermo Fisher Scientific, Tokyo, Japan). TaqMan Gene Expression Assays of haptoglobin (Hp; Rn00561393_m1) and glyceraldehyde 3-phosphate dehydrogenase (GAPDH; Rn99999916_s1) were used. Haptoglobin mRNA levels were normalized using GAPDH mRNA as a housekeeping gene.

### Histological analysis

Kidneys, livers, and spleens were harvested and fixed in 10% paraformaldehyde, and sections were cut from the paraffin-embedded tissue. Sections were stained with hematoxylin and eosin (HE) staining, periodic acid-schiff (PAS) staining, and Berlin blue (BB) staining. In the sections of kidneys stained with HE, tubulointerstitial lesion was histologically examined. Tubulointerstitial lesion score was quantified as follows: 0, normal; 0.5, small focal areas; 1, involvement of < 10% of the cortices and outer medullae; 2, 10–25% involvement of the cortices and outer medullae; 3, 25–75% involvement of the cortices and outer medullae; 4, extensive damage involving more than 75% of the cortices and outer medullae. Twenty fields of view were observed, and the average was taken as Tubulointerstitial lesion score^53–55^. In the sections of kidneys stained with PAS, glomerular sclerosis, tubular damage and arteriolosclerosis were histologically examined. Glomerular sclerosis was quantified as follows: 0, intact glomerulus; 1, 0–25% sclerosing glomerulus; 2, 25–50% sclerosing glomerulus; 3, 50–75% sclerosing glomerulus; 4, 75–100% sclerosing glomerulus. 50 glomeruli were scored, and the average was taken as Glomerular sclerosis score^55–57^. Tubular damage was quantified as follows: 0, no lesion; 1, very mild focal dilatation of tubules; 2, larger number of dilated tubules with widening if interstitium; 3, fairly extensive dilatation or tubules with cystic formation and widening of interstitium; 4, complete atrophy of tubules. Tissue injury in the medullary rays and inner stripe was separately scored for each section. Twenty fields of view were observed, and the average was taken as Tubular injury score^58^. Arteriolosclerosis was quantified as follows: 1, normal; 2, mild fibrous hypertrophy of the vascular wall is recognized and diagnosed as arteriole sclerosis; 3, infiltration of plasma components is observed in the submucosa from the submucosa to the muscle layer, but the cell wall proliferation in the vessel wall is not significant; 4, the lumen is mostly occluded, marked proliferation of the muscular layer, adventitial cells is observed. Ten blood vessels were judged, and the average was taken as Arterial Injury Score^59^. The specimens for microscopy were prepared by a pathology technician, and the microscopic scoring of the kidney sections was carried out by E. M., who was unaware of the treatment groups.

### Transmission electron microscopy of kidney

The tissue was pre-fixed with 4% paraformaldehyde and 5% glutaraldehyde, fixed with 2% aqueous osmium tetroxide solution, dehydrated with 50–100% gradual ethanol, embedded with propylene oxide and epoxy resin, and cured for 48 h or longer in a thermostatic chamber. The resin was cut thinly and placed on grids with a supporting film to perform uranium-lead staining and observed with transmission electron microscope (JEM-1400Plus, JEOL Ltd., Tokyo, JAPAN).

In the electron micrographs, mitochondrial damage in the renal proximal tubular epithelial cells on ferroptosis was evaluated using the percentage of mitochondria area in cytoplasm and the percentage of mitochondria with outer mitochondrial membrane rupture in the total number of mitochondria^60^. The pictures of electron micrographs were taken randomly by a pathology technician, and the microscopic scoring of the kidney sections was carried out by E. M., who was unaware of the treatment groups.

### Statistical analysis

All statistics were analyzed using the JMP Pro Version 14.2.0 software (SAS Institute Inc.). All values are reported as the means ± SEM. Statistical analysis was performed using unpaired Student t test, Wilcoxon test, or one-way analysis of variance followed by Tukey–Kramer test as appropriate. The associations between two parameters were analyzed using Pearson’s correlation coefficient. *p* values < 0.05 were considered statistically significant.
